# What happened to the patients? Care trajectories for persons with a delayed hospital discharge during wave 1 of COVID-19 in Ontario, Canada; a population-based retrospective cohort study

**DOI:** 10.1371/journal.pone.0309155

**Published:** 2024-09-27

**Authors:** Sara J. T. Guilcher, Yu Qing Bai, Walter P. Wodchis, Susan E. Bronskill, Laleh Rashidian, Kerry Kuluski

**Affiliations:** 1 Leslie Dan Faculty of Pharmacy, University of Toronto, Toronto, Ontario, Canada; 2 ICES, Toronto, Ontario, Canada; 3 Institute of Health Policy, Management, and Evaluation, Dalla Lana School of Public Health, University of Toronto, Toronto, Ontario, Canada; 4 Trillium Health Partners, Mississauga, Institute for Better Health, Ontario, Canada; 5 Sunnybrook Research Institute, Toronto, Ontario, Canada; Center for Primary Care and Public Health: Unisante, SWITZERLAND

## Abstract

During the initial wave of coronavirus disease of 2019 (COVID-19), patients were rapidly discharged from acute hospitals in anticipation of an expected influx of patients with COVID-19. Patients that were no longer receiving acute medical care but were waiting for their next destination (i.e., delayed hospital discharge) were particularly affected. The objectives of this study were to examine the impact of COVID-19 onset on healthcare utilization and mortality among those who experienced delayed discharge from acute care. We conducted a population-based retrospective cohort study using linked administrative data. We included persons discharged from acute care who experienced a delayed hospital stay between April 1, 2019 and September 30, 2020. The onset of COVID-19 was the exposure (March 1, 2020), while the period of April 1, 2019 to February 29, 2020 was considered as a comparator. Primary outcomes included healthcare utilization and mortality following discharge, stratified by care setting (homecare, inpatient rehabilitation or long-term care). Multivariable logistic, zero-inflated Poisson regressions, and Cox proportional hazard models were used to examine the impact of COVID-19 on outcomes while adjusting for covariates. Those discharged home were more likely to receive homecare and physician visits within 30 days during COVID-19. The type of visits examined included both in-person as well as virtual visits. Individuals discharged to inpatient rehabilitation experienced lower rates of general physician visits but higher rates of specialist and homecare visits. Patients discharged to long-term care were significantly less likely to receive a physician visit following COVID-19, and significantly more likely to be readmitted within 7-days. There were no significant differences in mortality irrespective of discharge destination during the two time periods. Overall, the onset of the initial wave of COVID-19 significantly impacted healthcare utilization among those with a delayed discharge but varied depending on destination, with those in long-term care being most impacted.

## Introduction

The onset of coronavirus disease of 2019 (COVID-19) substantially and rapidly changed how healthcare was being delivered across many countries. In Canada, delivery of healthcare dramatically changed in all sectors, including acute care, rehabilitation, long-term care, primary care, and homecare. Hospitals across the country quickly adopted measures, such as cancelling non-urgent procedures to reduce in-patient occupancy in anticipation of the potential surge of patients with COVID-19. Primary and specialist care were also impacted with many physicians quickly pivoting to virtual care rather than in-person visits [1]. Homecare was impacted during the initial waves, with an initial 25% decrease in new assessments [2], and many long-term care settings were not receiving additional patients from acute care or other settings [3].

Efforts to reduce acute care occupancy in the early months of COVID-19 directly impacted individuals who had finished their acute medical care and were waiting for their next care setting. Patients often referred to as those with delayed discharge, who would typically wait in acute inpatient hospitals for their next point of care, were identified as a priority for discharge during the pandemic in order to reduce in-patient occupancy and reduce risk of transmission of COVID-19 [4–6]. A recent study showed that delayed discharge rates in acute inpatient hospitals did not significantly change during the early COVID-19 pandemic [7], but the study did not consider post-discharge care trajectories, including healthcare utilization, outcomes, and overall experiences. Patients with a delayed discharge are mostly older adults with frailty, who are already considered a disproportionately vulnerable population [7–11]. Older adults discharged home, especially those with frailty, would likely require timely access to care including primary or specialist care and homecare services to support acute, chronic, and daily living needs [12].

COVID-19 significantly impacted how care was delivered in the community and may have increased adverse outcomes for adults who are older with more complex medical and social needs. Therefore, it is important to understand the post-acute outcomes during early COVID-19 which will inform health system performance for future crises. The present study had the following main objectives: to examine healthcare utilization and mortality rates, stratified by destination, among those who experienced a delayed discharge during the first wave of the COVID-19 pandemic in Ontario, Canada.

## Methods

### Study design and setting

We conducted a retrospective cohort study using linked administrative data from ICES (formerly known as the Institute for Clinical Evaluative Sciences) Toronto, Ontario (www.ices.on.ca). ICES is an independent, non-profit research institute funded by an annual grant from the Ontario Ministry of Health (MOH) and Ministry of Long-Term Care (MLTC). Ontario is Canada’s most populous province with more than 14.8 million residents. As a province within Canada that is governed by the Canada Health Act, Ontario provides universal medical coverage to residents for medically necessary services, including access to emergency departments, inpatient and outpatient hospitals, physicians, and some homecare services (though homecare services are considered extended health care services where each province or territory decides on the type and scope of care that they publicly fund) [13].

### Population

Persons with a delayed discharge from an acute care hospital in Ontario, Canada between April 1, 2019 and September 30, 2020 were included. Persons needed to be discharged alive to be included in the study, and could be discharged home (with or without homecare), to inpatient rehabilitation, or to long-term care. Persons discharged (transferred) to another acute inpatient setting were excluded from the analyses.

### Population and data sources

Datasets at ICES were linked using unique encoded identifiers. These data are valid and reliable, as described by numerous published studies [14–16]. We captured hospitalizations as well as procedures and diagnoses that occurred in hospital using the Canadian Institute of Health Information (CIHI) and Discharge Abstract Database (DAD). We identified records of emergency department visits using the National Ambulatory Care Reporting System (NACRS) and records of outpatient physician visits and physician specialty information using the Ontario Health Insurance Plan (OHIP) database. The Continuing Care Reporting System and Long-Term Care Database provided information on residents in complex continuing care and long-term care. Homecare was captured using the Home Care Database (HCD). We used numerous data sources to determine comorbidity, such as the Ontario Asthma Dataset, Congestive Heart Failure Dataset, Chronic Obstructive Pulmonary Disease Dataset, Ontario Hypertension Dataset, Ontario Diabetes Dataset, Ontario Rheumatoid Arthritis Dataset, and the Ontario Dementia Database. The Ontario Registered Persons Database provided demographic and mortality information [17–23]. The Ontario Drug Benefits Claims Database (ODB) was used to capture prescription drug claims for those 65 years of age or older, receiving social assistance (Ontario Works, Ontario Disability Benefits Plan), or receiving support for high-cost prescription drugs.

### Exposure

The primary exposure was COVID-onset as of March 1, 2020.

### Outcomes

Our main outcomes of interest included healthcare utilization and mortality in the 7-, 30- and 90-days following discharge. Healthcare utilization included emergency department visits, acute care readmissions, physician visits (overall, specialists, general and family physicians; 30- and 90-day, including in-person and virtual), and homecare visits (30- and 90-day). Both in-person and virtual physician visits by a general or family physician or specialty physician were included. Homecare visits were only examined for persons discharged home and/or post acute rehabilitation stay. We investigated outcomes stratified by discharge destination which included long-term care, homecare (including supportive living, with or without supports), and inpatient rehabilitation. Patients who had a long-term care or inpatient rehabilitation admission within 90 days post discharge of the index admission were grouped into the long-term care or inpatient rehabilitation category, respectively.

### Other variables

We described characteristics of the population by demographic information (age, sex), socioeconomic status (neighbourhood income), geography (rurality residence) and clinical status prior to admission date (comorbidities and previous drug claims). Neighbourhood income quintiles were calculated using census and postal code information. We determined urban and rurality residential location using the Rurality Index of Ontario. This index ranges from 0 to 100 and considers population factors and distance to referral centres. Rural locations have a score greater than or equal to 40 [24]. Using a validated multimorbidity algorithm at ICES, comorbidities were classified into 16 possible conditions, which included: acute myocardial infarction, asthma, arthritis, depression, diabetes, cancer, chronic coronary syndrome, cardiac arrythmia, congestive heart failure, chronic obstructive pulmonary disease, dementia, hypertension, renal failure, rheumatoid arthritis, and stroke [25]. We used the ODB database to capture records of prescription medications dispensed to individuals insured through the provincial drug plan in the year prior to the admission. Individuals are eligible for drug coverage if they are 65 years of age or older, reside in long-term care homes, receive homecare services, have high prescription medication costs compared to their net household income or receive social financial assistance through Ontario Works or Ontario Disability Benefits Plan.

Acute care admission characteristics (corresponding with the discharge date) included the type of admission (planned, unplanned), clinical category (surgical, medical), length of stay, frailty (decline in function in several organ systems) [26], and hospital harm [27]. Length of stay was captured in days and categorized as intensive care unit (ICU), acute, and alternate level of care (ALC). Alternate level of care is used to identify patients that no longer require the resource intensity that is provided in their current care setting [28]. Frailty was measured using a Hospital Frailty Risk Score (<5 low risk, 5–15 moderate risk, >15 high risk) [26]. The Hospital Frailty Risk Score was derived from the International Statistical Classification of Diseases and Related Health Problems Canadian Version (ICD-10-CA) codes and has been shown to be a valid measure of frailty [26]. Hospital harm is defined by CIHI as a hospitalization in which at least one unintended occurrence of a potentially preventable event occurs. The ICD-10-CA codes were used to identify and categorize harm into four major categories: 1) healthcare-/medication-associated conditions (e.g., pressure injuries, wrong medications); 2) healthcare-associated infections (e.g., surgical site infections); 3) patient accidents (e.g., falls); and 4) procedure-associated conditions (e.g., post-operative bleeding) [27].

### Statistical analysis

We used descriptive statistics to compare the demographic and clinical characteristics of patients discharged after a delay pre-COVID-19 and after the onset. Standardized differences were used to compare the populations as large sample sizes can result in statistical significance of trivial differences. Standardized differences are the difference in the mean of a variable between two groups, divided by the standard variation of that variable. We considered differences unimportant when below 10% (0.1) [29]. Multivariable logistic and zero-inflated Poisson regression models examined the change in health care utilization following COVID-19 onset, adjusting for covariates. Cox proportional survival models compared 30- and 90-day mortality. Survival time was examined using Kaplan-Meier survival curves. Models were stratified by destination (home, inpatient rehabilitation, and long-term care). Sensitivity analyses were conducted to omit persons who had COVID-19 and died during the observation window. All analyses were conducted at ICES using SAS Enterprise Guide 7.1 (SAS Institute Inc., Cary, NC, USA; www.sas.com).

### Ethics approval

As a prescribed entity under Ontario’s privacy legislation, ICES is authorized to collect and use healthcare data for the purposes of health system analysis, evaluation, and decision support. Secure access to these data is governed by policies and procedures that are approved by the Information and Privacy Commissioner of Ontario. The use of data was authorized under section 45 of Ontario’s *Personal Health Information Protection Act* [30], which does not require review by a Research Ethics Board.

## Results

### Patient and hospital stay characteristics

We identified 58788 patients from April 1, 2019 to September 30^th^ 2020 and 17504 patients between March 1, 2020 to September 30, 2020 who experienced a delayed discharge (see **Table 1**). In comparing the profile of patients before and after the onset of COVID-19, there were few meaningful differences of sociodemographic or clinical characteristics. In both time periods, most patients experiencing a delayed discharge were older (75 years or more), of female sex, living in neighbourhoods with lower income, and experienced multiple chronic conditions (5 or more).

**Table 1:** Comparison of patients with a delayed discharged by time period (pre vs onset of COVID-19), Ontario, Canada^a^.

Characteristics	Pre COVID-19 April 1, 2019-Feb 29, 2020	First Wave of COVID-19 Pandemic; March 1-September 30 2020	Standardized Difference (Pre vs. Onset)
**# of Unique Patients**	**N = 41284**	**N = 17504**	
**Length of Stay** Mean (SD)			
Total (days)	33.12(43.33)	25.78(24.16)	0.21
Intensive Care Unit (days)	2(9.35)	1.65(6.57)	0.04
Acute (days)	15.85(20.04)	13.38(13.84)	0.14
Delayed Discharge (days)	17.27(34.95)	12.4(18.6)	0.17
**Age**	** **	** **	
Mean (SD)	76.78(14.56)	76.79(14.78)	0.00
Median (IQR)	80(70–87)	80(69–87)	
**Sex**			
Female	22694(54.97)	9701(55.42)	0.01
Male	18590(45.03)	7803(44.58)	
**Neighbourhood Income**			
Q1 (Low)	11581(28.05)	4944(28.24)	0.00
Q2	9401(22.77)	3977(22.72)	0.00
Q3	7804(18.9)	3276(18.72)	0.00
Q4	6436(15.59)	2811(16.06)	0.01
Q5 (High)	6062(14.68)	2496(14.26)	0.01
**Rural**			
No	37496(90.82)	15860(90.61)	0.01
Yes	3788(9.18)	1644(9.39)	
**Comorbidities**			
0	745(1.8)	340(1.94)	0.01
1	1397(3.38)	598(3.42)	0.00
2	2657(6.44)	1155(6.6)	0.01
3	4216(10.21)	1801(10.29)	0.00
4	5598(13.56)	2398(13.7)	0.00
5+	26671(64.6)	11212(64.05)	0.01
**# of Unique Drugs Dispensed Prior to Admission**^b^ Mean (SD)	12.28(7.11)	12.11(6.98)	0.02
**Hospital Harm** ^c^			
# Harm Admissions	8419(20.39)	3413(19.5)	0.02
Care/Medications	4913(11.9)	1977(11.29)	0.02
Infections	4303(10.42)	1759(10.05)	0.01
Patient Accidents	554(1.34)	213(1.22)	0.01
Procedure	1025(2.48)	389(2.22)	0.02
**Hospital Frailty Score** Mean (SD)	8.5(5.37)	8.57(5.29)	0.01
**Hospital Frailty**			
Low risk (< 5)	12106(29.32)	4971(28.4)	0.02
Moderate (5–15)	24205(58.63)	10420(59.53)	0.02
High risk (> 15)	4973(12.05)	2113(12.07)	0.00
**Discharge Destination Post Index, n (%)**	** **	** **	
Long-term care	9002(21.81)	3118(17.81)	0.10
Home without support	14523(35.18)	6641(37.94)	0.06
Home with support	17759(43.02)	7745(44.25)	0.02

Abbreviations: SD = standard deviation; IQR = interquartile range

^a^Comparisons conducted upon discharge for index hospital admission

^b^Number of unique drugs dispensed in the year prior to index admission, only includes drugs dispensed among those who are eligible for the Ontario Drug Benefit plan

^c^Hospital Harm as per the Canadian Institute for Health Information Hospital Harm Index. We considered differences unimportant when below 10% (0.1)

There were differences in the acute care length of stay and the number of days with a discharge delay, with fewer days in hospital during COVID-19 (standardized differences >0.1). The median number of days in acute care (length of stay) was 10 days (IQR: 5–18) in the pre-COVID-19 period, compared to a median of 9 days during the pandemic (IQR: 5–16). Average length of stay also decreased from 15.8 days (SD = 20.0) pre-COVID-19 to 13.4 days (SD = 13.9) during the first COVID-19 wave (**Table 1**). For patients experiencing a delayed discharge, the median number of delayed days remained unchanged before and after COVID-19 (IQR: 3–15 pre-COVID-19, vs IQR: 2–14 during COVID-19).

### Impact of COVID-19 on healthcare utilization and mortality

**Table 2** provides a descriptive overview of healthcare utilization and mortality by destination among patients who experienced a delayed discharge. The number of physician visits post-discharge were generally higher when comparing the pandemic period to pre-pandemic for those who went home, and lower for those who went to long-term care (standardized mean differences ≥ 0.1), with visits not substantially changed for those discharged to inpatient rehabilitation. The average rate of emergency department visits and acute readmissions did not change substantially compared to prior to the COVID-19 pandemic (standardized mean differences < 0.1). Zero-inflated Poisson regression models were conducted to examine the impact of COVID-19 on emergency department visits, physician visits and homecare visits, after adjusting for covariates. Multivariate logistic regression models were used to examine the impact of COVID-19 on hospital readmissions, after adjusting for covariates (see **Tables 3** and **4**).

**Table 2:** Healthcare utilization and mortality following a delayed discharged in acute care, stratified by discharge destination and time period (pre vs onset of COVID-19), Ontario, Canada.

	Home (with or without support)			Inpatient Rehabilitation			Long-Term Care^a^		
	n = 21164			n = 25504			n = 12120		
Outcomes	Pre^†^	Post/Onset ^††^	Std. Diff	Pre	Post/Onset	Std. Diff	Pre	Post/Onset	Std. Diff
**Mortality post index discharge, n (%)**									
Within 30 days	912 (6.28)	440 (6.63)	0.01	1578 (8.89)	677 (8.74)	0.01	814 (9.04)	322 (10.33)	0.04
Within 90 days	1955 (13.46)	927 (13.96)	0.01	2685 (15.12)	1104 (14.25)	0.02	1510 (16.77)	584 (18.73)	0.05
**Acute Hospital Re-admissions**									
Within 7 days post discharge (index admission), Mean (SD)	0.07 (0.27)	0.07 (0.27)	0.01	0.03 (0.18)	0.03 (0.17)	0.01	0.04 (0.19)	0.05 (0.22)	0.05
Within 30 days post discharge (index admission), Mean (SD)	0.21 (0.48)	0.21 (0.48)	0.00	0.12 (0.36)	0.12 (0.35)	0.00	0.11 (0.35)	0.13 (0.37)	0.04
**Emergency Department Visits**									
Within 30 days post index discharge, Mean (SD)	0.21 (0.67)	0.19 (0.72)	0.03	0.08 (0.33)	0.08 (0.33)	0.02	0.11 (0.39)	0.09 (0.36)	0.05
**Physician Visits 30 Days Post Index Discharge**									
**Primary care physician visits**									
Mean (SD)	1.04 (1.63)	1.47 (2.06)	0.23	1.92 (3.86)	1.91 (3.69)	0.00	2.39 (3.22)	1.99 (2.78)	0.13
Median (IQR)	1 (0–1)	1 (0–2)		0 (0–2)	0 (0–2)		1 (0–3)	1 (1–2)	
**Specialist visits**									
Mean (SD)	0.6 (1.07)	0.71 (1.22)	0.10	0.81 (1.53)	0.86 (1.57)	0.03	0.43 (1.05)	0.32 (0.83)	0.12
Median (IQR)	0 (0–1)	0 (0–1)		0 (0–1)	0 (0–1)		0 (0–1)	0 (0–0)	
**Physician Visits 90 Days Post Index Discharge**									
**Primary care physician visits**									
Mean (SD)	2.5 (3.56)	3.32 (4.44)	0.20	4.47 (7.78)	4.55 (7.24)	0.01	5.41 (6.09)	4.46 (4.92)	0.17
Median (IQR)	2 (0–3)	2 (1–4)		2 (1–4)	2 (1–5)		3 (2–6)	3 (2–5)	
**Specialist visits**									
Mean (SD)	1.65 (2.48)	1.82 (2.66)	0.07	2.23 (3.07)	2.45 (3.24)	0.07	1.11 (2.21)	0.75 (1.53)	0.19
Median (IQR)	1 (0–2)	1 (0–3)		1 (0–3)	2 (0–3)		0 (0–2)	0 (0–1)	
**Homecare visits**									
**Within 30 days post index discharge**									
Mean (SD)	15.46 (16.14)	15.28 (16.41)	0.01	1.89 (4.43)	2 (4.65)	0.03			
Median (IQR)	9 (1–30)	9 (0–30)		0 (0–1)	0 (0–2)				
**Within 90 days post index discharge**									
Mean (SD)	38.3 (41.9)	37.81 (42.35)	0.01	15.32 (23.57)	15.78 (23.97)	0.02			
Median (IQR)	20 (2–72)	20 (2–70)		3 (0–22)	3 (0–23)				

^**†**^Pre COVID-19: April 1 2019-Feb 29, 2020

^**††**^Post-COVID March 1-September 30

Abbreviations: Std. Diff = standardized difference; SD = standard deviation; IQR = interquartile range; GP/FP = general practitioner/family practitioner

^a^ If patients were admitted to long-term care within 90 days post discharge then they were included in long-term care group.

Standardized differences were used to compare the populations admitted before and after COVID-19 onset. We considered differences unimportant when below 10% (0.1)

**Table 3:** Impact of the first wave of COVID-19 on health outcomes among patients with a delayed discharge in acute care, stratified by discharge destination, adjusting for covariates.

Outcome	Odds Ratio^b^ or Relative Risk^c^, 95%CI by Discharge Destination		
	Home (N = 21,164)	Inpatient Rehabilitation (N = 25,504)	Long-term Care (N = 12,120)
**Mortality** ^**a**^			
30 days	1.04 (0.93–1.17)	1.04 (0.95–1.14)	1.12 (0.98–1.28)
90 days	1.03 (0.95–1.12)	1.00 (0.93–1.07)	1.09 (0.99–1.20)
**Readmissions** ^**b**^			
7-day Readmissions	1.06 (0.94–1.19)	1.00 (0.85–1.19)	1.25 (1.02–1.53)
30-day Readmissions	1.00 (0.93–1.08)	1.06 (0.97–1.16)	1.11 (0.97–1.27)
**Emergency Department** ^**c**^			
30 days	1.07 (0.95–1.20)	1.10 (0.85–1.43)	0.87 (0.63–1.20)

^a^Cox Proportional Hazard Ratios

^b^Model: Multivariate Logistic Regressions

^c^ Model: Zero-Inflated Poissons Regressions

Covariates: Age, sex, income quintile, rurality, comorbidity, number of drugs previous year, frailty, hospital harm within index admission

**Table 4:** Impact of the first wave of COVID-19 on healthcare utilization, stratified by discharge destination, adjusting for covariates.

Utilization	Relative Risk^c^, 95%CI by Discharge Destination		
	Home (N = 21,164)	Inpatient Rehabilitation (N = 25,504)	Long-term Care (N = 12,120)
Homecare^c^			
30 days	1.01 (1.00–1.01)	1.02 (1.00–1.04)	
90 days	1.00 (0.99–1.00)	1.01 (1.00–1.01)	
**General Practitioner/** **Family Practitioner Visits** ^**c**^			
30 days	1.44 (1.39–1.49)	0.90 (0.89–0.92)	0.73 (0.71–0.75)
90 days	1.33 (1.30–1.35)	0.99 (0.98–1.01)	0.80 (0.78–0.82)
**Specialist Visits** ^**c**^			
30 days	1.17 (1.11–1.24)	1.06 (1.01–1.10)	0.85 (0.75–0.95)
90 days	1.10 (1.07–1.13)	1.09 (1.07–1.12)	0.72 (0.68–0.77)

^c^Model: Zero-Inflated Poissons

Covariates: Age, sex, income quintile, rurality, comorbidity, number of drugs previous year, frailty, hospital harm within index admission

### Patients discharged to home setting

Compared to pre-COVID-19, patients discharged home were more likely to visit a general or family practitioner during the first 30-days after discharge (RR = 1.17, 1.11–1.24), as well as 90-days post-discharge (RR = 1.10, 1.07–1.13) during the first wave of the COVID-19 pandemic. Patients discharged home were significantly more likely to have specialist visits during the first 30- and 90- days after discharge compared to the previous time-period (30-days RR = 1.17, 1.11–1.24, 90 days: 1.10,1.07–1.13). There were slightly more homecare visits in 30-days post discharge (RR = 1.01, 1.00–1.01) during the pandemic period compared to pre-pandemic. There were no identified differences due to COVID-19 in emergency department visits, readmissions, or mortality among this group.

### Patients discharged to an inpatient rehabilitation setting

By comparison, patients discharged to an inpatient rehabilitation setting were less likely to be visited by a general or family practitioner within the first 30 days following discharge from acute care during the COVID-19 period compared to pre-pandemic (RR = 0.90, 0.89–0.92). Conversely, there were higher rates of specialist visits compared to time periods before COVID-19 at 30-days (RR = 1.06, 1.01–1.10) and 90-days post-discharge (RR = 1.09, 1.07–1.12). The likelihood of receiving homecare visits in the inpatient rehabilitation setting was also significantly higher at 30-days (RR = 1.02, 1.00–1.04) and 90-days (RR = 1.01, 1.00–1.01) post-discharge during the pandemic period compared to pre-pandemic. There were no differences due to COVID-19 in emergency department visits, readmissions, or mortality among this group.

### Patients discharged to long-term care

Patients discharged to long-term care had a significantly lower likelihood of being seen by a general or family practitioner during the first 30-days and 90-days after discharge (RR = 0.73, 0.71–0.75), RR = 0.80, 0.78–0.82). Similarly, patients discharged to long-term care were significantly less likely to be seen by a specialist during the first 30- and 90- days after discharge compared to a period prior to COVID-19 (RR = 0.85, 0.75–0.95, RR = 0.72, 0.68–0.77, respectively). There were no differences in emergency department visits, or mortality among this group. However, 7-day readmissions were significantly higher among patients discharged to long-term care during COVID-19 compared to the previous time-period (OR = 1.25, 1.02–1.53); with no significant differences between time-periods for 30-day readmissions (OR = 1.11, 0.97–1.27).

## Discussion

In this population-based study using administrative health data, we examined the impact of the onset of COVID-19 on health care utilization and mortality rates following discharge from acute care among patients who experienced a delay. We identified that subsequent healthcare utilization varied by destination, with physician visits being the most impacted. Persons discharged home were more likely to have physician visits post-discharge during the initial onset of the pandemic compared to pre-pandemic. Conversely, persons discharged to long-term care were less likely to be seen by physicians and had higher readmission rates within the first 7 days post-discharge. However, we did not identify significant changes in short term mortality for any of the destinations.

Our finding of increased physician visits post discharge during the initial COVID-19 onset period for those discharged home may be a result of the efforts of primary care to address the shifting demands from acute to community. The rapid shift to virtual care that had occurred likely facilitated the ability to provide care for patients in the community [1, 31]. In the general population, physician visits initially dropped during the initial onset of COVID-19 but rebounded with the offering of virtual care [1, 32]. Numerous factors might be contributing to these findings, which include patient, clinician and system driven considerations. For example, persons initially going to inpatient rehabilitation may have more complex health and social needs rendering the need for more specialists during their rehabilitation stay. Virtual visits may have been less ideal to address medical assessment and treatment as well as rendering challenges if limited with access to technology. Furthermore, lower rates of virtual physician visits for some chronic diseases, such as hypertension, may stem from the necessity of physical examinations, as opposed to other diseases in which a comprehensive patient history via virtual visit may suffice for disease management. This is in line with findings measuring virtual access to care among patients with chronic conditions in Ontario [31].

Our findings regarding lower physician visits among patients in long-term care have been observed in other settings. CIHI also noted less medical care being provided in long-term care settings with fewer physician visits [33]. Patient profiles also seemed to differ during the pandemic period, which may have influenced physician visits. For example, one study found that older Ontarians living with dementia and Parkinson’s Disease were admitted to nursing homes at significantly lower rates during the pandemic, while also having lower rates of hospital visits and higher mortality compared to before the pandemic [34].

Future research is warranted to understand experiences with care transitions for patients and their care partners during the onset of COVID-19. Our study highlighted relatively positive system performance outcomes in those discharged home or to inpatient rehabilitation settings. More specifically, we did not observe an increase in mortality rates in response to reduced length of stay, patient, care partner and clinician experiences may have been less optimal. Specifically, though it appeared that people received services when discharged from hospital, it is unclear what the quality of those services were, particularly as the system became increasingly strained throughout the subsequent phases of the pandemic. Evidence suggests that policy changes driving rapid recruitment of homecare workers may have inadvertently resulted in recruitment of healthcare workers with insufficient training, with a detrimental impact on quality of care [35]. Research among patients with dementia living in long-term care homes found that up to 50% of healthcare providers reported a decrease in the perceived quality of care that was provided [36]. Staffing challenges and lower COVID-19 preparedness were also associated with a greater perceived decrease in quality of care in long-term care homes [36]. Identification of unmet healthcare needs among the adult Canadian population further suggests that quality of care was compromised during the COVID-19 pandemic [36, 37]. In addition, there is a need to understand factors that contributed to persons seeking hospital care during the pandemic. While there were policy changes restricting hospital admissions and procedures, patient level factors may have also contributed to the likelihood of seeking care (e.g., anxiety related to care provided in emergency and acute care settings) [38]. To further unpack care experiences, there is a need for qualitative and/or mixed-methods research. In addition, development of interventions to improve patient follow-up after acute care, and particularly in the setting of long-term care may ensure that these patients are receiving the care they need. A more fulsome understanding of experiences may provide an opportunity to study structures, processes, and outcomes around care transitions and consequences of different discharge scenarios, especially among long-term care settings. Since some hospitals were able to rapidly reduce occupancy (including delayed discharge rates) during the pandemic, understanding the lived experiences of patients, care partners, healthcare providers, and system administrators can be used to inform optimal care transitions and policies going forward in the post-pandemic period.

## Limitations

There are several limitations which should be acknowledged. Firstly, the data reflects delayed discharge rates during the first wave of COVID-19 and not subsequent waves. Health systems evolved in response efforts with each wave and as such, we cannot generalize that delayed discharge rates remained consistent for subsequent waves. Secondly, the data represents population level delayed discharge rates within Ontario, and cannot be generalized to other provinces or territories in Canada, since every province and territory had unique policies to address health system planning and delivery during the pandemic. However, the province of Ontario presents a unique opportunity for exploring the impact of COVID-19 to inform practice and policy for future health care crises. Administrative databases in Ontario hold health data for the full population, and are not limited to a particular subset of the population. This increases generalizability, particularly to settings such as other Western countries that responded similarly to the outbreak of COVID-19 [39].

## Conclusion

Overall, the onset of COVID-19 significantly impacted healthcare utilization among those with a delayed discharge but varied depending on destination. Those discharged to the community had higher physician visits and homecare visits, suggesting that primary and community care resources mobilized to address the acute needs of individuals. However, those discharged to long-term care had higher readmission rates within 7 days post discharge. Future research is warranted to understand the extent to which care needs were met for patients and their care partners.
